# Patients who received sleeve gastrectomy have lower plasma osteopontin levels than those who did not

**DOI:** 10.1016/j.clinsp.2024.100352

**Published:** 2024-04-03

**Authors:** Doğan Öztürk, Arzu Or Koca, Müge Keskin, Bülent Öztürk, Esra Fırat Oğuz, Turan Turhan, Hakan Buluş

**Affiliations:** a University of Health Sciences, Ankara Atatürk Sanatoryum Education and Research Hospital, Department of General Surgery, Ankara, Turkey; b University of Health Sciences, Dr. Abdurrahman Yurtaslan Ankara Onkoloji Education and Research Hospital, Department of Endocrinology and Metabolism, Ankara, Turkey; c University of Health Sciences, Ankara City Hospital, Department of Endocrinology and Metabolism, Ankara, Turkey; d University of Health Sciences, Ankara City Hospital, Department of Medical Biochemistry, Ankara, Turkey

**Keywords:** Sleeve gastrectomy, Osteopontin, Hepatocyte growth factor

## Abstract

**Background:**

The aim of this study was to compare metabolic parameters, plasma Osteopontin (OPN) and Hepatocyte Growth Factor (HGF) levels between Sleeve Gastrectomy (SG) patients in their 6^th^ post-operation month and healthy control patients.

**Methods:**

Height, weight, Body Mass Index (BMI) and laboratory parameters of 58 SG patients aged 18‒65 years (Group 1) and 46 healthy control patients (Group 2) were compared. In addition, preoperative and postoperative sixth-month BMI and laboratory parameters of the patients in Group 1 were compared.

**Results:**

The mean age and gender distributions of the groups were similar (p > 0.05). Mean BMI was 28.9 kg/m^2^ in Group 1 and 27 kg/m^2^ in Group 2 (p < 0.01). While plasma HGF levels were similar between both groups, plasma OPN levels were higher in Group 2 (p < 0.001). Fasting plasma glucose, total cholesterol, triglyceride, fasting plasma insulin and insulin resistance values were higher in Group 1, while alanine aminotransferase and aspartate aminotransferase levels were higher in Group 2 (p < 0.05). There was a strong correlation between plasma HGF and OPN levels in Group 1, but not in Group 2 (Rho = 0.805, p < 0.001).

**Conclusion:**

OPN and HGF are promising biomarkers that can be used to better understand and detect problems related to obesity. The fact that patients in the early post-SG period had lower plasma OPN and similar plasma HGF compared to non-surgical patients of similar age and gender with higher BMI may be another favorable and previously unknown metabolic effect of SG.

## Highlights

• Obesity causes higher plasma OPN levels and higher expression of OPN in adipose tissue.
• OPN and HGF are promising biomarkers that can be used to detect problems related to obesity.
• Patients in the early post-SG period had lower plasma OPN and similar plasma HGF compared to nonsurgical patients.
• After bariatric surgery, despite down-regulation of local OPN expression in adipose tissue, a significant increase in circulating OPN concentrations occurs

## Introduction

Certain proteins and hormones are now known to potentially lead to the discovery of promising biomarkers and therapies for metabolic disorders associated with overweightness and obesity. Osteopontin (OPN) and Hepatocyte Growth Factor (HGF) are two of the molecules that have been widely investigated in recent years. The effect of bariatric surgery, which causes significant weight loss, on plasma levels of these molecules and their metabolic consequences is currently a popular area of research.

Osteopontin is a combination of the words “osteo” meaning “bone”, and “pontine” meaning “bridge”, and it is the first extracellular matrix protein identified in bone tissue. OPN is also found in saliva, milk, fat, bile, dentin layer of teeth, kidneys, brains, bone marrows, endothelial cells, smooth muscle cells, skeletal muscle cells, and ganglia of the inner ear. It is the main component of the bone matrix and has various positive biological roles, such as increasing T-cell and macrophage count in inflammation, accelerating wound healing, and preventing kidney stones. However, it is also involved in biological processes that can be described as negative, such as OPN-induced inflammation playing a role in insulin resistance, and the fact that OPN release increases during tumor development and metastasis.^1^ As a proinflammatory cytokine, it is also associated with an increased risk of cardiometabolic disease.^2^

In an era when research is focused on obesity and related diseases, which are increasing inexorably all over the world, the effects of OPN, a proinflammatory mediator, on obesity are remarkable. Obesity causes higher plasma OPN levels and higher expression of OPN in adipose tissue. Diet-induced weight loss decreases circulating OPN levels.^3^ After bariatric surgery, despite the down-regulation of local OPN expression in adipose tissue, a significant increase in circulating OPN concentrations occurs. It is not clear whether these changes are related to alterations in bone metabolism or weight loss.^4^

HGF is a molecule that plays a pivotal role in metabolic disorders and particularly modulates inflammatory response. HGF was initially identified as a factor in liver regenerative circulating but was later found to be a protein that plays a variety of roles in many different tissues. HGF, a hepatokine, has been shown to be synthesized in human adipocytes. Increased HGF synthesis in adipocytes affects the pathogenesis of insulin resistance and related obesity.^5^

The aim of this study was to compare metabolic parameters, Plasma Osteopontin (OPN), and Hepatocyte Growth Factor (HGF) levels between Sleeve Gastrectomy (SG) patients in their sixth post-operation month and healthy control patients.

## Materials and methods

The study included 58 patients aged between 18 and 65 years who were followed up in the General Surgery Clinic of Health Sciences University Ankara Atatürk Sanatoryum Training and Research Hospital and who had undergone an SG operation six months prior (Group 1) and 46 healthy control patients who had no diagnosis of any chronic disease (Group 2).

Plasma OPN (ng/mL), HGF (pg/mL), C-Reactive Protein (CRP; mg/dL), Fasting Blood Glucose (FBG; mg/dL), fasting insulin (mIU/L), glycated Hemoglobin (HbA1c; %), creatinine (mg/dL), Alanine Aminotransferase (ALT; mg/dL), Aspartate Aminotransferase (AST; mg/dL), Alkaline Phosphatase (ALP; IU/L), Gamma-Glutamyltransferase (GGT; IU/L), direct bilirubin (mg/dL), indirect bilirubin (mg/dL), albumin (g/dL), Calcium (Ca; mg/dL), Phosphorus (P; mg/dL), 25-hydroxycholecalciferol (Vitamin D; ng/mL), Parathyroid Hormone (PTH; pg/mL), Total Cholesterol (TC; mg/dL), Triglyceride (TG; mg/dL), sodium (mEq/L), potassium (mmoL/L) levels of the patients were recorded. Additionally, Homeostasis Model Assessment of Insulin Resistance (HOMA-IR) values were calculated.^6^

Venous blood samples were collected into vacutainer tubes and centrifuged at 1300 × g for 10 minutes. Separated sera were aliquoted into Eppendorf tubes and stored at -80°C until analysis. Human OPN and HGF levels were measured with ELISA kits (ELK Biotechnology, Wuhan, China) using the quantitative sandwich enzyme immunoassay technique. Optical Density (OD) was measured spectrophotometrically utilizing a microplate reader at a wavelength of 450 nm. The OD value is proportional to the concentration of human OPN and HGF levels. The concentration of the samples was calculated by comparing the OD of the samples to the standard curve. The detection range of the OPN assay (catalog no: ELK1047- lot no: 20343235507) was 0.63–40 ng/mL and the sensitivity of the assay was 0.263 ng/mL. Intra- and interassay precision were < 8% and < 10%, respectively. The detection range of the Hepatocyte Growth Factor assay (catalog no: ELK1032-lot no: 20343235470) was 31.25‒2000 pg/mL and the sensitivity of the assay was 12.3 pg/mL. Intra- and interassay precision were < 8% and < 10%, respectively.

Height, weight, Body Mass Index (BMI), and data regarding the diagnosis of Type 2 Diabetes Mellitus (T2DM) were recorded for all participants. In addition, preoperative height, weight, BMI, and laboratory analysis data of the patients in Group 1 were recorded.

Exclusion criteria were a history of rheumatologic disease and/or malignancy, impaired thyroid function evidenced by tests, use of anti-obesity drugs, having undergone a bariatric surgery other than SG, complications after SG, acute infection, elevated CRP levels, not having suppressed cortisol levels evident by preoperative dexamethasone suppression tests, renal dysfunction, known liver pathologies, a history of cerebral operation, use of corticosteroids and pregnancy.

### Ethics statements

Ethics committee approval was obtained from Health Sciences University Ankara Atatürk Sanatoryum Training and Research Hospital Clinical Research Ethics Committee (2012-KAEK-15/2733). Verbal and written informed consent were obtained from all participants. The study was conducted in accordance with the ethical principles of the Declaration of Helsinki.

### Statistical analysis

The data of the study was analyzed by SPSS 25.0 (IBM®, New York, USA). The findings were expressed as frequency and percentages. The Shapiro-Wilk test was used to determine the parametric distribution of the variables. The variables with or without parametric distribution are presented as mean ± standard deviation and median (min-max), respectively. The paired samples *t*-test and Wilcoxon signed-rank test were used to compare repeated measurements of variables. Parametric and non-parametric variables of different groups were compared using the independent samples *t*-test and Mann-Whitney *U* test, respectively. Spearman Correlation analysis was performed to determine variables associated with HGF and OPN. The Correlation between HGF and OPN was illustrated with a scatterplot graphic for both groups. A statistical significance of p < 0.05 was considered significant.

## Results

The study included 58 participants in Group 1 and 46 participants in Group 2. The mean age and gender distributions of the groups were similar (p > 0.05). Mean BMI was 28.9 kg/m^2^ in Group 1 and 27 kg/m^2^ in Group 2 (p < 0.01). Excess weight loss (%) for Group 1 is 81.04 ± 12.51, and sufficient weight loss frequency is 98.3 (57/58). FBG, total cholesterol, TG, HOMA-IR, and insulin levels were higher in Group 1, while ALT and AST levels were higher in Group 2 (p < 0.05) (Table 1).

**Table 1:** Demographic and clinical characteristics of the groups.

		Group 1 (n = 58)	Group 2 (n = 46)	p
***Mean ± Standard Deviation or Median (minimum‒maximum)***				
***Demographic data***				
Age (year)		37.45 ± 10.11	40.24 ± 7.33	0.119
Gender (n/%)	Female	43 (74.1)	38 (82.6)	0.348
	Male	15 (25.9)	8 (17.4)	
BMI (kg/m^2^)		28.9 (24.2‒35.6)	27.0 (22.0‒41.5)	**<0.01**
***Clinical data***				
Excess weight loss		81.04±12.51	‒	
Sufficient weight loss (n/%)		57 (98.3)	‒	
Frequency of T2DM (%)		15 (25.9)	‒	
FBG (mg/dL)		91.5 (74.0‒178.0)	80.0 (68.0‒142.0)	**<0.001**
HbA1c (%)		5.3 (3.7‒9.7)	4.9 (4.2‒5.5)	0.181
Creatinine (mg/dL)		0.7 (0.5‒1.1)	0.8 (0.4‒1.3)	0.263
Sodium (mEq/L)		140.0 (132.0‒146.0)	139.0 (136.0‒144.0)	0.407
Potassium (mmol/L)		4.2 (2.8‒5.1)	4.3 (2.9‒5.4)	0.212
ALT (IU/L)		18.5 (10.0‒70.0)	27.0 (12.0‒56.0)	**0.014**
AST(IU/L)		22.5 (2.0‒59.0)	36.0 (17.0‒74.0)	**<0.001**
GGT(IU/L)		32.5 (9.0‒81.0)	37.0 (8.0‒66.0)	0.463
Direct bilirubin (mg/dL)		0.3 (0.1‒1.8)	0.4 (0.2‒3.6)	0.141
Albumin (g/dL)		3.8 (2.7‒4.6)	3.6 (2.8‒4.8)	0.857
Ca (mg/dL)		9.1±0.1	8.4±0.7	**<0.001**
P (mg/dL)		3.5±0.4	3.7±0.4	0.325
ALP (IU/L)		44.0 (8.0‒113.0)	38.0 (19.0‒88.0)	0.146
Vitamin D (ng/mL)		25.8±13.6	23.6±9.2	0.555
PTH (pg/mL)		42.2±11.5	44.7±7.9	0.128
TC (mg/dL)		155.2±56.5	144.3±31.9	**<0.001**
TG (mg/dL)		179.0 (54.0‒1041.009	152.0 (97.0‒245.0)	**0.015**
Insulin (µIU/mL)		17.3±8.4	8.9 ± 2.0	**0.017**
HOMA-IR		3.7 (1.6‒18.7)	1.6 (1.2‒3.9)	**<0.001**

ALP, Alkaline Phosphatase; ALT, Alanine Aminotransferase; AST, Aspartate Aminotransferase; BMI, Body Mass Index; Ca, Calcium; FBG, Fasting Blood Glucose GGT, Gamma-Glutamyltransferase; HbA1c, Glycated Hemoglobin; HOMA-IR, Homeostatic Model Assessment for Insulin Resistance; P, Phosphorus; PTH, Parathyroid Hormone; TC, Total Cholesterol; TG, Triglycerides; T2DM:, Diabetes mellitus Type 2.

**Fig. 1:**
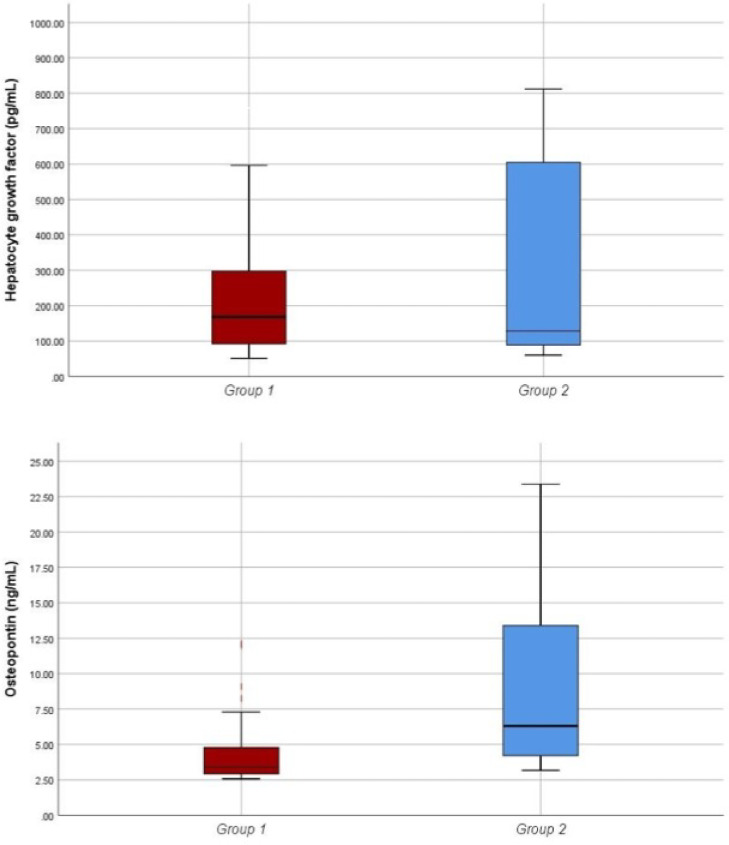
HGF and OPN levels of Group 1 and Group 2. HGF, Hepatocyte Growth Factor; OPN, Osteopontin.

HGF levels of the two groups were statistically similar (p = 0.649), but OPN levels were significantly higher in Group 2 than in Group 1 (p < 0.001) (Table 2) (Fig. 1).

**Table 2:** The comparison of hepatocyte growth factor and osteopontin levels of the groups.

	Group 1 (n = 58)	Group 2 (n = 55)	p
HGF (pg/mL)	172.5 (51.4-758.8)	128.8 (60.1-811.7)	0.649
OPN (ng/mL)	3.4 (2.6-12.1)	6.3 (3.2-23.4)	**<0.001**

HGF, Hepatocyte Growth Factor; OPN, Osteopontin.

Preoperative and postoperative sixth-month BMI values and laboratory parameters of the patients in Group 1 were compared. The mean BMI of the patients decreased from 45.5 kg/m^2^ preoperatively to 28.9 kg/m^2^ postoperatively (p < 0.001). Preoperative HbA1c, ALT, albumin, ALP, ALP, TG, insulin, and HOMA-IR levels were significantly higher than postoperative levels, while preoperative vitamin D, PTH and TC levels were found to be lower (Table 3).

**Table 3:** Comparison of body mass index and laboratory parameters of the bariatric surgery group before and after surgery.

	Before surgery	After surgery	p
BMI (kg/m^2^)	45.5 (40.0‒61.6)	28.9 (24.2‒35.6)	**<0.001**
FBG (mg/dL)	97.0 (47.0‒217.0)	91.5 (74.0‒178.0)	0.082
HbA1c (%)	5.8 (4.1‒10.2)	5.3 (3.7‒9.7)	**<0.01**
Creatinine (mg/dL)	0.7 (0.4‒1.1)	0.7 (0.5‒1.1)	0.840
Sodium (mEq/L)	140.0 (136.0‒187.0)	140.0 (132.0‒146.0)	0.969
Potassium (mmoL/L)	4.1 (3.2‒4.9)	4.2 (2.8‒5.1)	0.971
ALT (IU/L)	29.0 (9.0‒118.0)	18.5 (10.0‒70.0)	**<0.01**
AST(IU/L)	21.5 (11.0‒63.0)	22.5 (2.0‒59.0)	0.870
GGT(IU/L)	35.5 (8.0‒72.0)	32.5 (9.0‒81.0)	0.320
Direct bilirubin (mg/dL)	0.2 (0.1‒5.9)	0.3 (0.1‒1.8)	0.059
Albumin (g/dL)	4.1 83.1‒4.8)	3.8 (2.7‒4.6)	**<0.01**
Ca (mg/dL)	9.3 ± 0.5	9.1 ± 0.1	0.220
P (mg/dL)	3.5 ± 0.5	3.5 ± 0.4	0.798
ALP (IU/L)	44.0 (8.0‒113.0)	29.0 (11.0‒108.0)	**<0.01**
Vitamin D (ng/mL)	25.8 ± 13.6	29.4 ± 1.8	**0.019**
PTH (pg/mL)	42.2 ± 11.5	53.8 ± 22.1	**<0.01**
Total cholesterol (mg/dL)	155.2 ± 56.5	182.3 ± 30.1	**<0.01**
Triglyceride (mg/dL)	179.0 (54.0‒1041.009	126.5 (3.8‒318.00)	**<0.01**
Insulin (µIU/mL)	16.0 (7.3‒57.0)	5.7 (2.5‒22.0)	**<0.001**
HOMA-IR	3.7 (1.6‒18.7)	1.3 (0.5‒7.2)	**<0.001**

ALP, Alkaline Phosphatase; ALT, Alanine Aminotransferase; AST, Aspartate Aminotransferase; BMI, Body Mass Index; Ca, Calcium; FBG, Fasting Blood Glucose; GGT, Gamma-Glutamyltransferase; HbA1c, Glycated Hemoglobin; HOMA-IR, Homeostatic Model Assessment for Insulin Resistance; P, Phosphorus; PTH, Parathyroid Hormone; TC, Total Cholesterol; TG, Triglycerides.

**Fig. 2:**
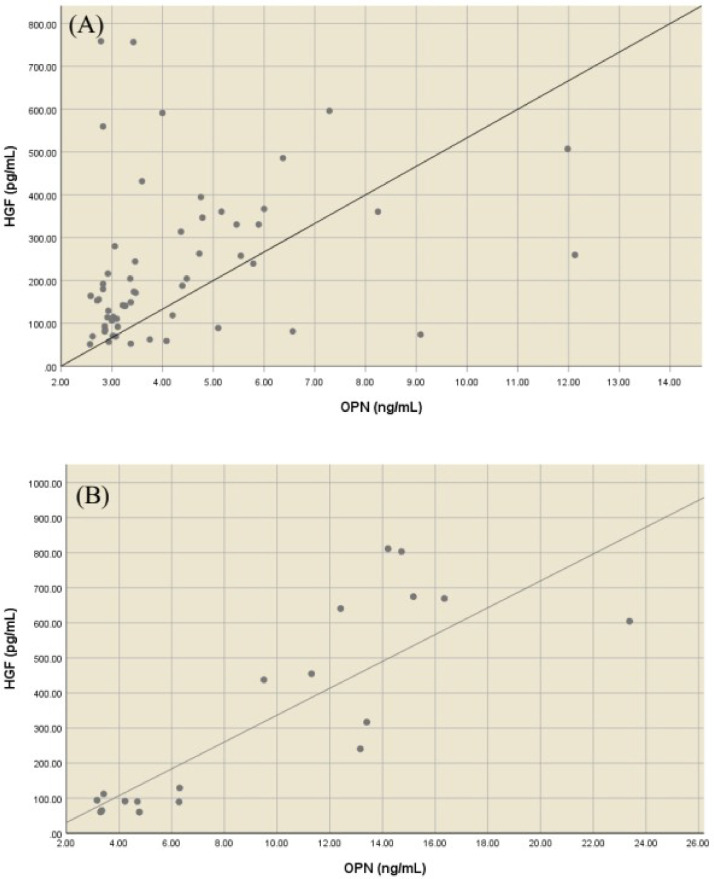
(A) Scatterplot graphic of HGF and OPN of Group 1. (B) Scatterplot graphic of HGF and OPN of Group 2.

As a result of correlation analysis between HGF and OPN values and other variables in Group 1, a weak positive correlation between HGF and OPN (Rho = 0.395, p < 0.01) and a weak negative correlation between HGF and GGT (Rho = -0.338, p < 0.01) were found. There was a weak positive correlation between OPN and AST (Rho = 0.270, p = 0.040) and a weak negative correlation between OPN and ALP (Rho = -0.363, p < 0.01). HGF and OPN were not correlated with age, gender, BMI, T2DM frequency, insulin, HOMA-IR, thyroid function test results, ALT, Ca, P, albumin, ALP, PTH, and 25-OH vitamin D levels (p > 0.05). There was a strong positive correlation between HGF and OPN (Rho = 0.805, p < 0.001) and a weak positive correlation between HGF and vitamin D levels (Rho = 0.374, p = 0.010). No other demographic, clinical, or laboratory parameter was correlated with HGF and OPN in Group 2. Scatterplots of HGF and OPN levels of the groups are shown in Fig. 2.

## Discussion

In this study, it was shown that overweight individuals in their sixth-month post-SG had no difference in plasma HGF levels and had lower plasma OPN levels than overweight individuals who had not undergone surgery.

The 2017 study by Gómez-Ambrosi et al. demonstrated elevated plasma OPN levels in overweight and obese individuals for the first time.^3^ The relationship between OPN and obesity has aroused great interest, especially in the last two decades, and has been examined in many studies on obesity and related diseases.^7^, ^8^, ^9^, ^10^, ^11^, ^12^ The relationship between OPN and obesity is based on the effect of OPN on increased insulin resistance.^13^^,^^14^ Obese individuals with T2DM have significantly higher plasma OPN levels than obese normoglycemic individuals.^3^ Notably, plasma OPN levels have been shown to increase after bariatric surgery, independent of increased insulin sensitivity and weight loss. According to Schaller et al., this is probably due to the rapid change in bone metabolism after bariatric surgery.^10^ The case group of the current study was unique in that it included participants who had undergone SG operation six months before. However, the preoperative/postoperative plasma OPN changes could not be evaluated due to the lack of preoperative plasma OPN level data.

As was expected in the initial hypothesis, it was found that plasma OPN levels were higher in the control group, who had lower BMI, and not in the case group with higher BMI. It was noteworthy that the control group had high plasma OPN levels even though they had lower BMI. This finding is thought to be related to the bariatric surgeries the case group underwent. It is known that bariatric surgery is not only a restrictive surgery but also a metabolic surgery.^15^^,^^16^ The authors suggest that the low plasma OPN levels observed in Group 1 composed of overweight individuals in the early postoperative period may be another favorable metabolic effect of bariatric surgery, independent of weight loss. This finding highlights the accuracy of the terminology “metabolically healthy obese”. However, this result may also be due to the fact that the control group had a high body fat percentage, despite having a BMI in the range of 25‒30 kg/m^2^.

OPN is a glycoprotein that affects a wide range of tissues. It is likely that isoforms of OPN are responsible for its different effects across the various tissues. Sarosiek et al. showed a close association of the OPNc isoform with obesity, whereas the other isoforms, OPNa and OPNb, were not found to be associated with obesity.^17^ The difference in plasma OPN levels between the groups may be related to the dominance of different isoforms of OPN in the participant population. Further studies are needed to uncover the differences between the effect of OPN isoforms.

In an animal study by Kiefer et al., it was found that antibody-mediated neutralization of OPN's effects resulted in significant improvement in obesity-related metabolic dysfunction.^18^ Low plasma OPN levels have a protective effect against obesity-related hepatic steatosis.^19^ Plasma OPN is strongly associated with hepatic enzymes, which are often elevated in obese patients due to higher liver fat. In the current study, a weak negative correlation was found between HGF and GGT and OPN and ALP levels, and a weak positive correlation was found between OPN and AST levels. These findings were consistent with the results of prior studies.^3^^,^^11^^,^^20^^,^^21^

OPN is a molecule highly sensitive to changes in the energy cycle. Therefore, changes in plasma OPN after weight loss and exercise are not unusual. Acute cycling exercise reduces plasma OPN, which leads to long-term improvements in cardio-metabolic health.^22^ Moderate weight loss can lead to significant changes in plasma OPN levels.^23^ Although data on their preoperative plasma OPN levels did not exist, the case group of the current study achieved significant weight loss and a favorable change in metabolic parameters within six months of surgery, and their mean BMI decreased from 45.5 kg/m^2^ to 28.9 kg/m^2^ after SG. The low plasma OPN of the present case group may be related to rapid adipocyte loss. OPN receptors are expressed in adipocytes, and exogenous OPN significantly inhibits insulin-stimulated 2-deoxyglucose uptake in adipocyte cell cultures. However, exogenous OPN also inhibits adipogenesis. The paracrine effects of OPN in adipocyte tissue, although not yet fully elucidated, appear to be a down regulator of adipogenesis. OPN has even been called the “enemy of adipocytes”.^24^

There are conflicting results in the literature regarding plasma OPN changes after various bariatric surgical procedures. A study conducted on rats showed that plasma OPN decreased after SG, even though the rats continued to be fed a high-fat diet.^25^ In their study involving 24 participants, Komorowski et al. showed that plasma OPN levels of the participants increased after vertical banding surgery.^26^ In another study involving five patients with morbid obesity, the patients had elevated plasma OPN levels after bariatric surgery.^10^ The study of Katsogiannos et al. also demonstrated increased plasma OPN levels after Roux-en-Y Gastric Bypass (RYGB). Riedl et al. showed that laparoscopic adjustable gastric banding and RYGB patients had elevated OPN levels one year after surgery.^27^ Lancha et al. suggested that while RYGB increased circulating OPN levels, SG does not alter circulating OPN.^28^ There are several potential reasons for the conflicting results between this study and the literature. Firstly, OPN has multiple functions related to bone metabolism, cardiovascular disease, T2DM, and obesity. Therefore, plasma OPN levels are controlled by many factors. Other tissues and/or physiologic factors may affect plasma OPN levels more than adipose mass.^29^ However, rapid weight loss after bariatric surgical procedures causes significant changes in bone metabolism.^30^ Due to it being an important part of the bone matrix, rapid changes in OPN levels after bariatric surgery are to be expected. In addition, weight loss and metabolic effects caused by malabsorptive, and restrictive bariatric surgical procedures differ from each other.^28^ Moreover, dynamic changes that differ according to time after surgery continue to occur in the body. For example, bone metabolism changes in the first postoperative year are quite different compared to those in the third postoperative year.^17^ The factors mentioned above may explain the discrepancies in the data found in the literature.

Another proinflammatory molecule the authors evaluated in this study was HGF. Obesity is associated with elevated circulating HGF. Obese individuals have circulating HGF levels that are three times greater than those of non-obese individuals.^24^ A previous study demonstrated that perivascular adipose tissue had a much higher HGF secretion potential compared to subcutaneous and visceral adipose tissue, and that elevation in HGF was directly proportional to perivascular adipose tissue.^29^ Bariatric surgery is known to decrease plasma HGF due to its effect on weight loss.^30^ Since preoperative plasma HGF data of the patients did not exist, the authors could not examine plasma HGF changes in the case group. No difference in plasma HGF was found between the case and control groups, despite higher insulin resistance and BMI in the case group. This may be due to bariatric surgery decreasing plasma HGF levels independent of weight loss.

The main limitation of this study was that preoperative plasma OPN and HGF levels of patients undergoing SG were not recorded. Other limitations include the limited number of participants and the fact that only postoperative sixth-month laboratory parameters of the case group were evaluated. However, strengths of the study include the exclusion of confounding factors by using a wide range of exclusion criteria and demonstrating weakened inflammation associated with SG in a group that has a higher BMI than prior studies through biochemical markers.

## Conclusion

Obesity is a condition that cannot be classified and managed with a simple formula of weight/height^2^. Although studies on obesity, which has a very complex pathogenesis, mostly focus on bariatric surgical procedures and their effects, there are many other unknowns regarding obesity that are still waiting to be elucidated. The fact that patients in the early post-SG period had lower plasma OPN and similar plasma HGF compared to non-surgical patients of similar age and gender with higher BMI may be another favorable and previously unknown metabolic effect of SG. Like many other cytokines, the complex functions of OPN in adipocyte tissue are becoming more understood every day, as OPN is becoming a source of hope in better understanding, preventing, and treating obesity.

## Authors’ contributions

Idea/Concept: Doğan Öztürk, Arzu Or Koca; Design: Arzu Or Koca; Supervision/Consulting: Hakan Buluş, Turan Turhan; Data Collection and/or Processing: Doğan Öztürk, Bülent Öztürk, Esra Fırat, Oğuz; Analysis and/or Interpretation: Arzu Or Koca, Müge Keskin; Literature Review: Arzu Or Koca, Doğan Öztürk, Müge Keskin; Writing the Article: Arzu Or Koca; Critical Review: Hakan Buluş.

## Funding

There is no funding.

## Declaration of competing interest

The authors declare no conflicts of interest.
